# Novel Magnetic-to-Thermal Conversion and Thermal Energy Management Composite Phase Change Material

**DOI:** 10.3390/polym10060585

**Published:** 2018-05-27

**Authors:** Xiaoqiao Fan, Jinqiu Xiao, Wentao Wang, Yuang Zhang, Shufen Zhang, Bingtao Tang

**Affiliations:** State Key Laboratory of Fine Chemicals, Dalian University of Technology, Dalian 116024, China; fxq@mail.dlut.edu.cn (X.F.); xjq@dlut.edu.cn (J.X.); wentao_wang@mail.dlut.edu.cn (W.W.); zhangyuang@mail.dlut.edu.cn (Y.Z.); zhangsf01@sohu.com (S.Z.)

**Keywords:** superparamagnetic Fe_3_O_4_ nanocluster, magnetothermal conversion, phase-change material, thermal energy management

## Abstract

Superparamagnetic materials have elicited increasing interest due to their high-efficiency magnetothermal conversion. However, it is difficult to effectively manage the magnetothermal energy due to the continuous magnetothermal effect at present. In this study, we designed and synthesized a novel Fe_3_O_4_/PEG/SiO_2_ composite phase change material (PCM) that can simultaneously realize magnetic-to-thermal conversion and thermal energy management because of outstanding thermal energy storage ability of PCM. The composite was fabricated by in situ doping of superparamagnetic Fe_3_O_4_ nanoclusters through a simple sol–gel method. The synthesized Fe_3_O_4_/PEG/SiO_2_ PCM exhibited good thermal stability, high phase change enthalpy, and excellent shape-stabilized property. This study provides an additional promising route for application of the magnetothermal effect.

## 1. Introduction

Under the alternating magnetic field, magnetic nanoparticles can produce a large amount of heat energy by the magnetothermal effect as a result of Néel relaxation or Brownian relaxation [1,2,3,4]. More importantly, magnetic nanoparticles exhibit superparamagnetism when their size is reduced to a certain extent [5,6], which has relatively high magnetic susceptibility and no remanence or coercivity after removal of the magnetic field [7,8]. These advantages, coupled with magnetothermal effect of magnetic nanoparticles, provide various useful applications that range from cancer treatment [9,10] to magnetically triggered drug delivery [11,12]. However, the thermal energy from magnetothermal conversion of superparamagnetic materials was difficult to manage effectively due to the continuous magnetothermal effect [13,14,15].

Phase change material (PCM), a substance that changes its state (from liquid to solid, or solid to liquid) and provides latent heat as the temperature changes, is one of the most prospective thermal energy management materials [16,17,18,19,20,21]. These materials can store and release a large amount of heat energy at a small temperature change during phase transition [22,23,24,25,26,27,28,29,30,31,32,33,34,35]. If superparamagnetic Fe_3_O_4_ and PCM are combined, obtained composite will have dual functions of magnetothermal conversion and heat management.

**Figure 1:**
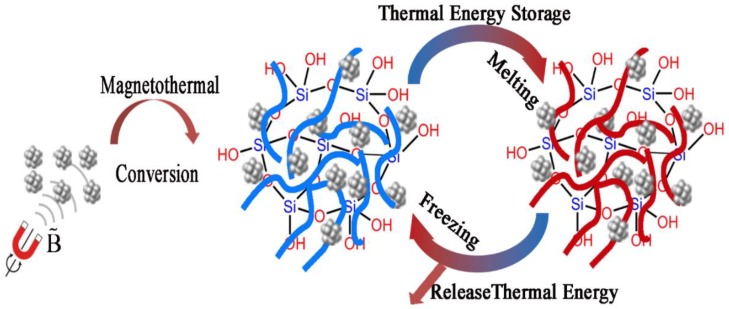
Schematic of magnetic-to-heat conversion and storage.

Based on the above idea, we reported a novel Fe_3_O_4_/PEG/SiO_2_ composite PCM via the facile doping of superparamagnetic nano Fe_3_O_4_ through in situ sol–gel method. Superparamagnetic nano Fe_3_O_4_ possess high saturation magnetization (Ms) and negligible remanence, and they were successfully combined with a form-stable PCM system in this work. The thermal energy dissipated by superparamagnetic nano Fe_3_O_4_ was stored by PCM in the way of phase transition (Figure 1). Thus, this strategy can effectively control the temperature of the magnetothermal conversion system by managing the thermal energy from magnetothermal conversion of superparamagnetic materials.

## 2. Materials and Methods

### 2.1. Materials

Iron (III) chloride anhydrous (FeCl_3_) was obtained from Xilong Chemical Company, Ltd., Shantou, China. Diethylene glycol (DEG) and Sodium acetate anhydrous (CH_3_COONa) were supplied by Guangfu Fine Chemical Research Institute, Tianjin, China. Ethylene glycol (EG) was obtained from Fuyu Fine Chemical Company, Tianjin, China. Trisodium citrate dihydrate (C_6_H_5_Na_3_O_7_·2H_2_O) was purchased from Damao Chemical Reagent Factory, Tianjin, China. Analytical grade polyethylene glycol (PEG, *M*_n_ = 6000) was purchased from Aladdin, Shanghai, China. Tetraethoxylsilane (TEOS, 98% purity) was provided from Sigma-Aldrich, St. Louis, MO, USA. All other reagents were of analytical grade.

### 2.2. Synthesis of Fe_3_O_4_/PEG/SiO_2_ Composite

The preparation of Fe_3_O_4_/PEG/SiO_2_ composite was divided into two procedures. The first procedure was the synthesis of superparamagnetic Fe_3_O_4_ nanoclusters. In this procedure, Fe_3_O_4_ nanoclusters were prepared using a typical solvothermal method. Firstly, FeCl_3_ (10 mmol) and Na_3_Cit (1 g) were added to a mixed solvent of DEG and EG (DEG:EG = 1:1, total volume = 80 mL) under mechanical stirring at 120 °C and dissolved to form a clear solution. Secondly, NaAc (50 mmol) was put in the clear solution and stirred vigorously for the next 1h. Then, the obtained homogeneous solution was transferred into a 100 mL of Teflon-lined stainless steel autoclave and heated to 200 °C. After maintaining this condition for 10 h, the sealed autoclave was cooled to room temperature. Finally, the obtained precipitate was separated from the mixed solvent by a magnet and rinsed with deionized water for several times.

**Figure 2:**
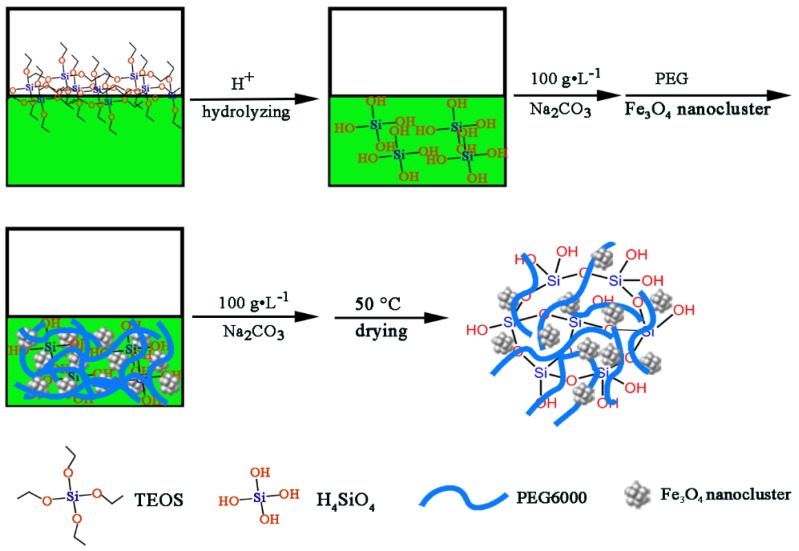
Schematic of the synthesis of Fe_3_O_4_/PEG/SiO_2_ composite.

In the second procedure, Fe_3_O_4_/PEG/SiO_2_ composite PCM was synthesized via a sol–gel method. Figure 2 illustrates a schematic of the preparation method of Fe_3_O_4_/PEG/SiO_2_ composite. A specified amount of TEOS was mixed with deionized water at a mole ratio of 1:10. After stirring for several minutes, 0.5 M of hydrochloric acid was dropped into the solution, and the pH of the solution was adjusted to 1–2. The hydrolysis reaction proceeded until the solution became clear. Subsequently, 100 g·L^−1^ of sodium carbonate solution was added to adjust pH to 5–6, and the melted PEG and Fe_3_O_4_ homogeneous aqueous solution was put in silica sol under stirring. Silica gel containing PEG and Fe_3_O_4_ was obtained after 100 g·L^−1^ of Na_2_CO_3_ solution was added to adjust the pH of the solution. The PCM whose PEG content was 80 wt % was obtained when the gel was dried in a vacuum drying oven at 50 °C.

### 2.3. Characterization

Scanning electron microscopy (SEM) images were obtained on a FEI Nova NanoSEM 450 apparatus. High-resolution transmission electron microscopy (HRTEM) was recorded on a FEI Tecnai G2 F30 microscope operating at 300 kV. X-ray powder diffraction (XRD) patterns of the products were performed on a Japan Rigaku D/Max 2400 automatic X-ray powder diffractometer equipped with Cu-Kα radiation (*λ* = 1.54178 Å) within 5–80°. FTIR spectra of the products were recorded using a Nicolet 460 FTIR spectrophotometer from 4000–600 cm^−1^. Thermogravimetric analyses (TGA) and differential scanning calorimeter (DSC) curves were performed by using a TA Instruments Universal Analysis 2000. All DSC measurements were both carried out from 5 °C to 80 °C at the heating/cooling rate of 10 °C/min and a N_2_ flow rate of 50 mL/min. All TG measurements were performed in N_2_ atmosphere and the range of heating is from room temperature to 700 °C. The M–H and ZFC–FC curves were measured with a Quantum Design MPMSXL-7 system. Digital photos were taken with a Nikon digital camera.

Magnetic-to-thermal conversion and energy storage test of the Fe_3_O_4_/PEG/SiO_2_ composites: the 1 g of composites were put into small bottles (internal diameter, 1.2 cm); a certain alternating magnetic field (1.36 MHz and 550 A·m^−1^) was applied to the composites with an alternating current generator; changing temperature of product was measured using a fiber sensor plugged into it.

## 3. Results and Discussion

### 3.1. Characterization of Superparamagnetic Fe_3_O_4_

**Figure 3:**
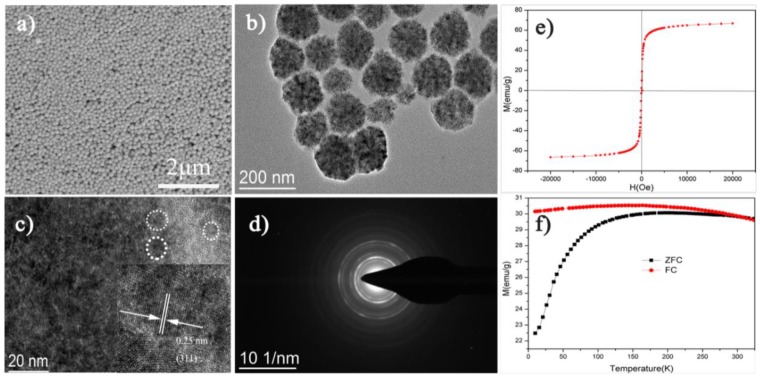
(**a**) SEM image of nano Fe_3_O_4_. (**b**,**c**) Low-and high-magnification TEM images of nano Fe_3_O_4_, the inset of (**c**) is lattice fringe images of 152 nm Fe_3_O_4_. (**d**) SAED pattern of the cluster in (**c**). (**e**) Hysteresis loops of the Fe_3_O_4_ nanoclusters at 300 K. (**f**) The ZFC-FC curves of nano Fe_3_O_4_ at 500 Oe.

Fe_3_O_4_ superparamagnetic nanoclusters were fabricated via a modified solvothermal method. The morphology and microstructure of nano Fe_3_O_4_ were investigated through SEM and TEM. The obtained results are shown in Figure 3a–d. Figure 3a,b revealed that the diameter of nanoclusters was about 159 nm on the average and the morphology of nano Fe_3_O_4_ was spherical. Lattice fringes were recorded for an isolated nanocluster, and whose diameter was 152 nm, as shown in the high-resolution TEM (HRTEM) image in Figure 3c. Obviously, a cluster consisted of many small primary particles according to this image. The measured distance between two adjacent planes in the same direction was 0.25 nm, which conformed to the lattice spacing of (311) planes of cubic magnetite. The selected-area electron diffraction (SAED) pattern recorded for the same particle was diffraction rings and revealed that nano Fe_3_O_4_ is polycrystalline. The diffraction spots were widened into narrow arcs, which indicated slight misalignments among the primary nanocrystals.

Mass magnetization of the as-prepared products was measured at room temperature (300 K) in an applied magnetic field, in which the cycle range of H was from −20 kOe to 20 kOe. Figure 3e indicated that Fe_3_O_4_ nanoclusters had a saturation magnetization (Ms) of 66.7 emu g^−1^ and negligible coercivity and remanence, suggesting that the obtained nanoclusters are superparamagnetic at room temperature. To further demonstrate the superparamagnetic behavior of nano Fe_3_O_4_ at room temperature, standard zero field cooling (ZFC) and field cooling (FC) curves were measured under a magnetic field of 500 Oe (Figure 3f). The maximum of the ZFC curve appeared at about 190 K, which was the superparamagnetic blocking temperature (*T*_b_) of the Fe_3_O_4_ nanoclusters [36], and the temperature illustrated a transition from a magnetically blocked state (at low temperature) to a superparamagnetic state (at high temperature). After this temperature, a bifurcation point was observed at around 260 K in the ZFC−FC curve, which is the irreversibility temperature where the ZFC magnetization curve departs from the FC curve.

### 3.2. Characterization of Fe_3_O_4_/PEG/SiO_2_ Composite

**Figure 4:**
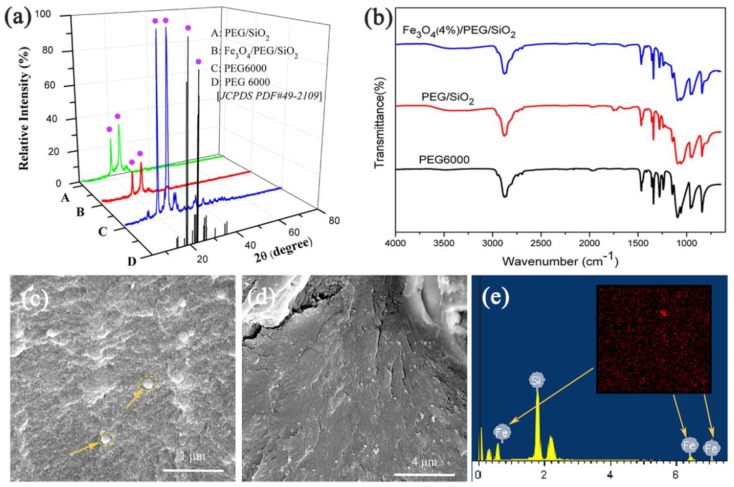
(**a**,**b**) The XRD patterns and FTIR spectra of PEG6000, PEG/SiO_2_, and Fe_3_O_4_/PEG/SiO_2_ composite. (**c**,**d**) SEM images of Fe_3_O_4_/PEG/SiO_2_ composite at different magnification times. (**e**) The corresponding EDS analysis.

X-ray diffraction (XRD) is an efficient means to investigate the crystallization property of materials. Figure 4a shows the XRD patterns of PEG/SiO_2_, Fe_3_O_4_/PEG/SiO_2_, and PEG6000. As shown in the picture, sharp and intense diffraction peaks were observed at approximately 18.9° and 23.1° for PEG/SiO_2_ and Fe_3_O_4_/PEG/SiO_2_ composite, respectively, similar to pure PEG6000. Therefore, SiO_2_ and Fe_3_O_4_ exerted no effect on the crystal structure of PEG6000 [37]. In addition, the lower intensity of peaks observed in XRD pattern of PEG/SiO_2_ and Fe_3_O_4_/PEG/SiO_2_ compared to PEG6000. Notwithstaning, PEG/SiO_2_ and Fe_3_O_4_/PEG/SiO_2_ composite still possessed good crystalline properties, which guaranteed the energy storage ability of the PCMs because thermal energy storage was closely related to the crystallization property of PCM [38].

Figure 4b shows the FTIR spectra of PEG6000, PEG/SiO_2_, and Fe_3_O_4_/PEG/SiO_2_ composites. The pure PEG6000 had the following characteristic absorption bands: (i) stretching vibration of O–H at 3430 cm^−1^, (ii) stretching vibration of CH_3_ at 2917 cm^−1^, (iii) stretching vibration of CH_2_ at 2889 cm^−1^, and (iv) symmetric stretching vibration of C–O–C at 1106 cm^−1^. Compared to that of pure PEG, the FTIR spectra of the synthesized Fe_3_O_4_(4%)/PEG/SiO_2_ and PEG/SiO_2_ included a symmetric stretching vibration of Si–OH in the range of 3032–3700 cm^−1^, asymmetric stretching vibration of Si–O–Si at 1050 cm^−1^, and bending vibration of SiO–H at 796 cm^−1^, besides all representative bands for pure PEG6000.

Figure 4c,d present SEM photographs of Fe_3_O_4_/PEG/SiO_2_ composite, in which the surface of PEG/SiO_2_ showed small Fe_3_O_4_ nanoclusters and their distribution is denoted by yellow arrows in Figure 4c. The result demonstrated that Fe_3_O_4_ superparamagnetic nanoclusters were loaded on the PCM. Moreover, the Fe_3_O_4_ nanoclusters were distributed uniformly on the surface of PCM in Figure 4d. EDS analysis in Figure 4e also demonstrated that ferrum (Fe) existed in the Fe_3_O_4_/PEG/SiO_2_ composite and was distributed uniformly.

### 3.3. Thermal Properties

**Figure 5:**
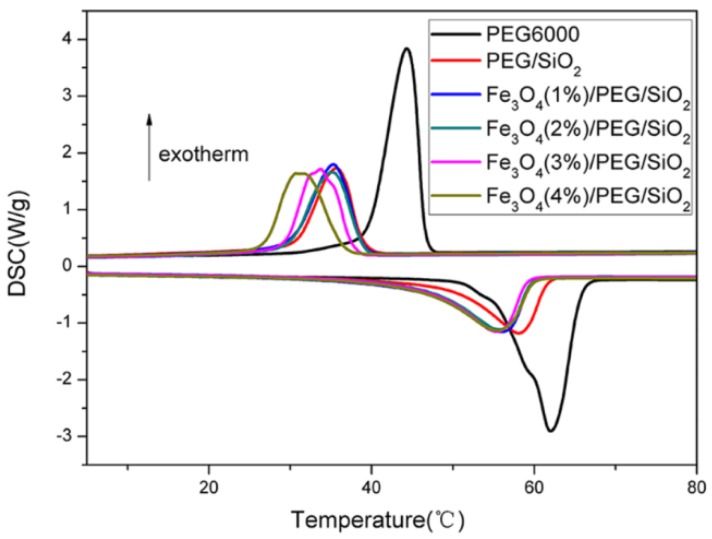
DSC curves of Fe_3_O_4_/PEG/SiO_2_ PCMs and PEG/SiO_2_ composite.

The thermal characteristic plays a critical role in the thermal management ability of PCMs. Figure 5 presents the DSC curves of PEG6000, PEG/SiO_2_, and Fe_3_O_4_/PEG/SiO_2_ with different Fe_3_O_4_ contents. The endothermic and exothermic enthalpy values and the phase transition temperatures obtained from the DSC curves are listed in Table 1. The endothermic and exothermic enthalpy values of the Fe_3_O_4_/PEG/SiO_2_ PCMs were 114–122 and 109–114 J/g, respectively. Enthalpies of fusion of composites are 162.4 J/g in theory because additive amount of PEG is about 80% of total mass of composites. Compared to the theoretical values, enthalpies of fusion of PEG/SiO_2_ and Fe_3_O_4_/PEG/SiO_2_ composites decrease by 25–30%, which is likely to be caused by the interference of SiO_2_ network with the crystallization of PEG, this phenomenon is consistent with the result reported for confinement effects of SiO_2_ framework on phase change of PEG [39]. They had a high phase-change enthalpy and heat storage capacity, although the PEG/SiO_2_ and Fe_3_O_4_/PEG/SiO_2_ composites showed a partial loss of latent heat compared with the pure PEG (Figure 5 and Table 1). Enthalpy of Fe_3_O_4_ (3%)/PEG/SiO_2_ with the average diameter of 95 nm (130.5 J/g) is higher than that of Fe_3_O_4_ (3%)/PEG/SiO_2_ with the average diameter of 159 nm (114.7 J/g). This is probably because that smaller size of doped Fe_3_O_4_ is more favorable to microphase separation and causes a more continuous PEG domain, which benefits crystallization of PEG in Fe_3_O_4_/PEG/SiO_2_ composites (Figure S1). In addition, the phase transition temperatures listed in Table 1 are close to that of pure PEG6000.

### 3.4. Shape-Stabilized Properties

**Figure 6:**
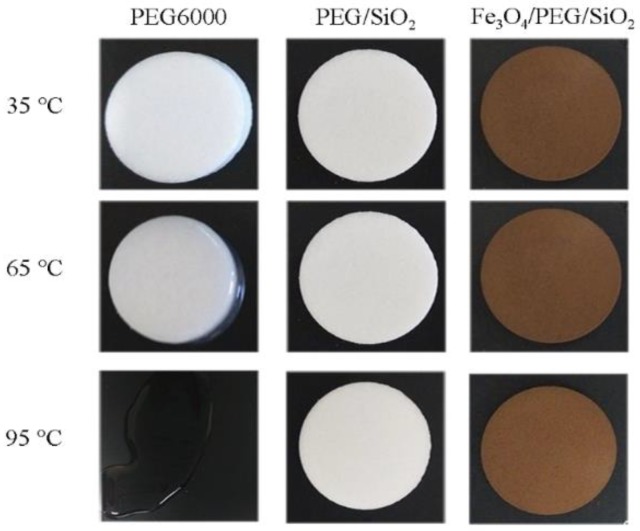
Photos of pure PEG, PEG/SiO_2_, and Fe_3_O_4_/PEG/SiO_2_ composites at different temperature.

Shape-stabilized properties are crucial aspects in the application of PCMs. Figure 6 shows digital photos of PCMs at different temperatures. The samples were placed in an oven at constant temperature. The changes in the macroscopic morphology of samples were observed and recorded by a digital camera immediately after 20 min. Pure PEG6000 appeared a melted ring and melted completely when it was placed in the oven for 20 min at 65 °C and 95 °C, respectively. However, the PEG/SiO_2_ and Fe_3_O_4_/PEG/SiO_2_ composites were shape-stabilized and did not show any leakage behavior at either 65 °C or 95 °C. This is because that the PEG is a long-chain polymeric PCM that can completely or partially interpenetrate the network of SiO_2_ gel. The SiO_2_ gel network can limit the leakage of PEG by capillary force and surface tension when PEG changes from solid to liquid [27,40]. Therefore, PCMs doped with superparamagnetic Fe_3_O_4_ possess an excellent shape-stabilized property.

### 3.5. Magnetic-To-Thermal Conversion and Thermal Energy Management Performance

**Figure 7:**
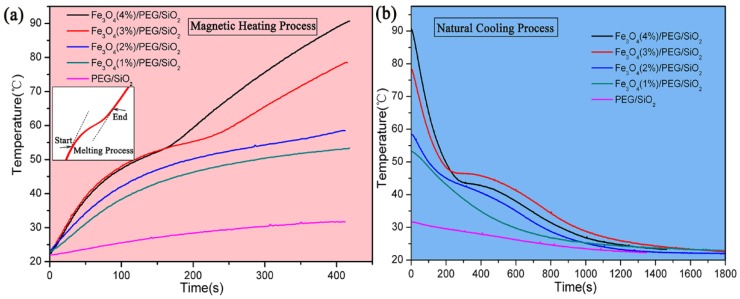
Magnetic-to-thermal energy conversion and thermal management curves of the samples (m = 1 g) under the alternating magnetic field (1.36 MHz, 900 A·m^−1^). (**a**) Curves of storing thermal energy in magnetic heating process, inset shows temperature change platform of phase change process. (**b**) Curves of releasing thermal energy in natural cooling process.

Synthesized Fe_3_O_4_/PEG/SiO_2_ composite PCMs exhibited excellent magnetothermal conversion and thermal energy management behavior under an alternating magnetic field. Figure 7 shows the magnetic-to-thermal energy conversion and thermal energy management curves of the Fe_3_O_4_/PEG/SiO_2_ composites. As shown in the magnetic heating curves, when the content of nano Fe_3_O_4_ in the composites increased, temperature rise rate increased—i.e., heating efficiency increased—so Fe_3_O_4_/PEG/SiO_2_ possess the best heating efficiency when additive amount of Fe_3_O_4_ is 4 wt %. After heating for 420s under the alternating magnetic field, the temperatures of the Fe_3_O_4_/PEG/SiO_2_ materials with Fe_3_O_4_ contents of 1% to 4% reached 53.4 °C, 58.5 °C, 78.6 °C, and 90.7 °C, respectively. There is a growth platform appeared between 42 °C and 57 °C for Fe_3_O_4_/PEG/SiO_2_ PCMs (2, 3, and 4%) when the alternating magnetic field was applied (inset of Figure 7). The times that Fe_3_O_4_/PEG/SiO_2_ PCMs (2, 3, and 4%) are in the growth platform stage of temperature are 120, 165, and 277 s, respectively. In this stage, thermal energy from magnetothermal conversion is stored in the way of latent heat. Fe_3_O_4_ (1%)/PEG/SiO_2_ did not have this growth platform because it had less magnetic particles, such that phase transition did not occur completely in the same period of time. Likewise, the phenomenon appeared in the process of natural cooling. Moreover, the temperature of PEG/SiO_2_ increased slowly, which is a reflection of the fact that PEG/SiO_2_ composite did not have the component of nano Fe_3_O_4_. In addition, heating rate increases and time of melting platform shortens, when the intensity of alternative magnetic field increases (Figure S2). Overall, combining with PCM can achieve effective management of thermal energy from magnetothermal conversion.

### 3.6. Reversible Stability

**Figure 8:**
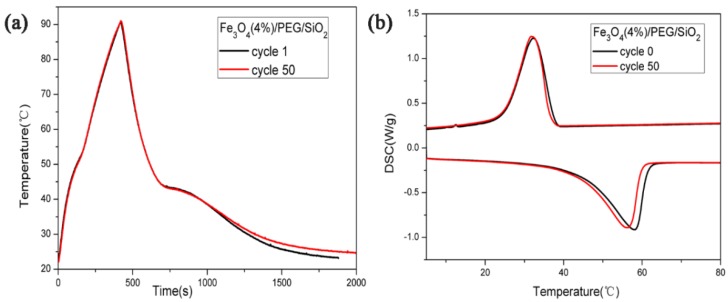
(**a**) Magnetic-to-thermal energy conversion curves of the Fe_3_O_4_/PEG/SiO_2_ composite before and after the 50 cycles. (**b**) DSC curves of the Fe_3_O_4_/PEG/SiO_2_ composite before and after the 50 cycles.

It is important for composite phase change materials that have good reversibility of energy storage and releasing when managing magnetothermal energy. Therefore, the magnetic-to-thermal conversion cycling tests were performed in this work. Figure 8a shows the results of the first and 50th cycles under the same conditions. The curve of the 50th cycle was nearly coincident with that of the first cycle. The DSC curves before and after the cycling test were also almost identical (Figure 8b). The peaks after the cycling test presented a very small shift compared with the peaks before the cycling test. These findings confirm that the synthesized Fe_3_O_4_/PEG/SiO_2_ materials possess high reversibility.

### 3.7. Thermal Stability

**Figure 9:**
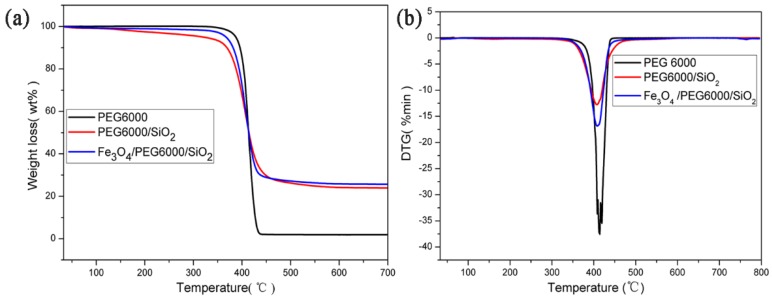
TG (**a**) and DTG (**b**) curves of PEG6000, PEG/SiO_2_, and Fe_3_O_4_/PEG/SiO_2_ composites.

Besides reversible stability, thermal stability is also vital for Fe_3_O_4_/PEG/SiO_2_ composite PCM. The TG and DTG curves in Figure 9 explain the thermal degradation behaviors of the PCMs. Both PEG/SiO_2_ and Fe_3_O_4_/PEG/SiO_2_ composites had a steep thermal degradation process that occurred between 325 °C and 476 °C, which is consistent with that of pure PEG and far above phase transition temperature. Furthermore, the weight losses of approximately 76.2 wt % and 75.4 wt % in composites were observed in TG curves from room temperature to 700 °C, which is nearly close to the theoretical content of PEG in composites (80 wt %, in accordance with the synthesis experiment). These results show that obtained PCM composites possess high thermal stability at phase transition temperature.

## 4. Conclusions

In conclusion, we designed and synthesized a novel composite of Fe_3_O_4_/PEG/SiO_2_ that can achieve effective thermal energy management for magnetic-to-thermal conversion process. The composite was fabricated by in situ doping of superparamagnetic Fe_3_O_4_ nanoclusters through a simple sol–gel method. Nano Fe_3_O_4_ effectively converted magnetic work into internal energy that was released into the surrounding environment in the form of thermal energy when applying alternative magnetic field, and the converted thermal energy was stored in the PCM and can be exploited. In addition, the synthesized Fe_3_O_4_/PEG/SiO_2_ composite demonstrated several outstanding characteristics, such as high thermal stability and excellent shape-stabilized properties, which meets the requirements for practical application. Thus, our composites exhibit the potential for realizing the conversion from electromagnetic energy to thermal energy, and moreover, management and control of the resulting thermal energy can also be realized by phase transition component in composites. This study will be a helpful strategy for management of magnetothermal energy, such as temperature control in magnetic hyperthermia.

## Figures and Tables

**Table 1:** Phase change behavior of pure PEG, PEG/SiO_2_, and Fe_3_O_4_/PEG/SiO_2_ composites with different Fe_3_O_4_ contents.

Sample	Phase Transition	Δ*H* (J·g^−1^)		*T*_r_ (°C)	
		Heating Cycle	Cooling Cycle	Heating Cycle	Cooling Cycle
PEG6000	Solid-liquid	203.0	197.0	62.0	44.3
PEG/SiO_2_	Form-stable	109.3	105.6	58.0	35.6
Fe_3_O_4_(1%)/PEG/SiO_2_	Form-stable	117.0	110.0	56.0	35.3
Fe_3_O_4_(2%)/PEG/SiO_2_	Form-stable	115.1	109.7	55.8	35.1
Fe_3_O_4_(3%)/PEG/SiO_2_	Form-stable	114.7	111.2	55.5	33.7
Fe_3_O_4_(4%)/PEG/SiO_2_	Form-stable	121.6	114.0	55.4	30.7

## Supplementary Materials

The following are available online at http://www.mdpi.com/2073-4360/10/6/585/s1, Figure S1: (a) SEM image of Fe_3_O_4_ nanoparticles with the average diameter of 95 nm. (b) DSC curves of Fe_3_O_4_(3%)/PEG/SiO_2_ composites with different sizes of doped Fe_3_O_4_ nanoparticles; Figure S2: The temperature evolution curves of Fe_3_O_4_(4%)/PEG/SiO_2_ at alternative magnetic field with different magnetic strengths.
