# Supplementary material for: Novel Magnetic-to-Thermal Conversion and Thermal Energy Management Composite Phase Change Material
Source: Polymers (Basel). 2018 May 27;10(6):585. doi: 10.3390/polym10060585 (PMC6404046; doi:10.3390/polym10060585)
Supplement: Supplementary file 1 [file polymers-10-00585-s001.docx]

Supplementary Materials: Novel Magnetic-to-Thermal Conversion and Thermal Energy Management Composite Phase Change Material

Xiaoqiao Fan, Jinqiu Xiao, Wentao Wang, Yuang Zhang, Shufen Zhang and Bingtao Tang*


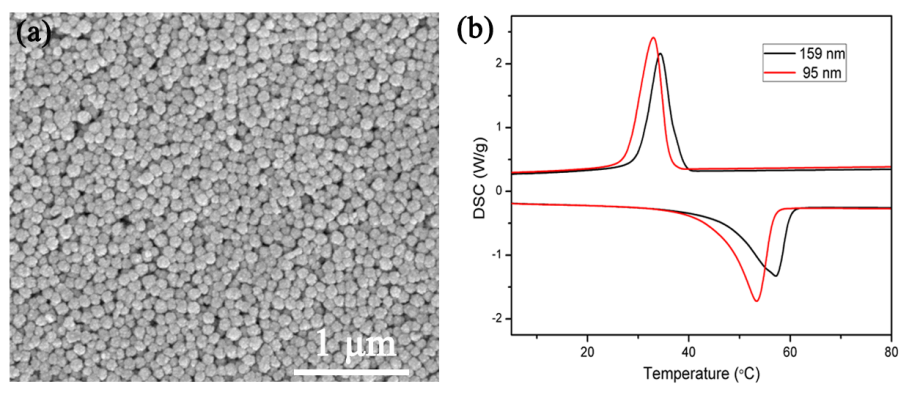


**Figure S1.** (a), SEM image of Fe_3_O_4_ nanoparticles with the average diameter of 95 nm. (b), DSC curves of Fe_3_O_4_(3%)/PEG/SiO_2_ composites with different sizes of doped Fe_3_O_4_ nanoparticles.


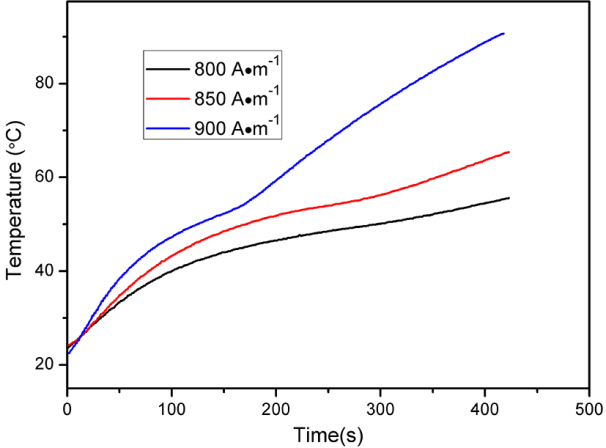


**Figure S2.** The temperature evolution curves of Fe_3_O_4_(4%)/PEG/SiO_2_ at alternative magnetic field with different magnetic strengths.
